# Genetic Characterization and Pathogenicity of a Triple‐Reassortant H1N1 Swine Influenza Virus Isolated in Guangdong

**DOI:** 10.1155/tbed/7175378

**Published:** 2026-04-10

**Authors:** Benkuan Li, Yu Wu, Yi Song, Hui Guo, Guangrun Qin, Limiao Lin, Haishen Zhao, Bohua Ren, Qunhui Li, Lianxiang Wang, Huihua Zhang

**Affiliations:** ^1^ School of Animal Science and Technology, Foshan University, Foshan, 528225, China, fosu.edu.cn; ^2^ Wen’s Food Group, Yunfu, 527400, China; ^3^ College of Veterinary Medicine, South China Agricultural University, Guangzhou, 510642, China, scau.edu.cn

**Keywords:** H1N1, molecular characterization, pathogenicity, phylogenetic analysis, swine influenza virus

## Abstract

An H1N1 subtype swine influenza virus (SIV) was isolated from nasal swabs collected from pigs with suspected swine influenza (SI) on a farm in Guangdong, China. The virus, designated A/Swine/Guangdong/JJK/2025(H1N1) (abbreviated as JJK), underwent whole‐genome sequencing and phylogenetic analysis, which confirmed that it is a triple‐reassortant strain. The hemagglutination (HA) and neuraminidase (NA) genes originated from the Eurasian avian‐like (EA) H1N1 lineage, the internal genes (PB2, PB1, PA, NP, and M) originated from the 2009 pandemic H1N1 lineage, and the NS gene originated from the triple‐reassortant H1N2 lineage. Each of the eight gene segments showed the highest homology to a different reference virus. Analysis of key amino acid substitutions identified multiple mutations in proteins, such as HA, PB2, and PB1, that are associated with enhanced mammalian adaptation, increased virulence, and modulation of host immune responses. Animal pathogenicity testing demonstrated that JJK was significantly pathogenic in female BALB/c mice, with an MLD_50_ of 10^4.67^ EID_50_/50 μL, and caused severe lung lesions with high viral loads. However, in piglets, infection elicited substantial increases in serum inflammatory cytokines (TNF‐α, IFN‐γ, IL‐6, and IL‐10) but produced only mild clinical signs and limited pulmonary inflammation. To address the need for continuous surveillance of these evolving lineages, this study characterizes the genetic features and differential pathogenicity of a reassortant H1N1 SIV in distinct animal models, providing essential evidence to support epidemiological surveillance, risk assessment, and integrated control strategies for SI in China.

## 1. Introduction

Swine influenza (SI) is an acute, highly contagious respiratory disease in pigs caused by the SI virus (SIV). Its primary clinical manifestations include anorexia, fever, coughing, and nasal discharge, with severe cases progressing to respiratory distress, such as dyspnea [1, 2]. Although infection with a single SIV strain typically results in low mortality, the disease is characterized by high morbidity and wide circulation within pig populations. Moreover, SIV infections frequently predispose pigs to secondary infections with other pathogens, which can increase mortality and cause substantial economic losses to the swine industry [3, 4]. Pigs are considered “mixing vessels” for influenza viruses [5]. Their respiratory epithelial cells express both SAα‐2,6‐Gal receptors, which are preferentially bound by human influenza viruses, and SAα‐2,3‐Gal receptors, which are preferred by avian influenza viruses. This dual receptor distribution enables pigs to serve as an efficient platform for genetic exchange and reassortment among influenza viruses from different species [6–8].

Multiple SIV subtypes, including H1, H3, and H5, have been reported worldwide, with H1N1 remaining the most prevalent. H1N1 subtype SIVs primarily include classical H1N1 (CS H1N1), Eurasian avian‐like H1N1 (EA H1N1), human‐origin H1N1, and the 2009 pandemic H1N1 (pdm/09 H1N1). After SI was first identified in 1918, CS H1N1 was isolated and continued to circulate in pig populations [9–11]. It later spread to Europe and Asia, becoming the dominant lineage [12] during that period. In 1979, a novel influenza virus containing all eight gene segments of avian origin was detected in Europe, providing clear evidence of cross‐species transmission from birds to pigs [13, 14]. During the 1990s, the EA H1N1 lineage entered Chinese swine herds and rapidly replaced CS H1N1, becoming the dominant lineage in China after 2005 [15, 16]. The 2009 H1N1 influenza pandemic profoundly reshaped the global influenza ecology. Following the emergence of pdm/09 H1N1, the virus spread widely in humans and repeatedly entered swine populations through reverse zoonotic transmission, substantially influencing the evolutionary dynamics of SIVs [17, 18]. These spillover events facilitated extensive genetic reassortment with circulating lineages such as EA H1N1 and CS H1N1 in pigs [19, 20]. Since 2016, reassortant EA H1N1 viruses have become the predominant SIV type in China [21–23]. In these viruses, internal genes (PB2, PB1, PA, NP) may be entirely or partially derived from pdm/09 H1N1, whereas the surface genes (hemagglutination [HA], neuraminidase [NA]) often retain characteristics of the EA or other lineages. These complex reassortment events have contributed to a highly genetically diverse SIV ecosystem in China [16]. H1N1 viruses circulating in Chinese pigs now exhibit a mosaic genomic structure with unprecedented diversity. This continuous evolution and reassortment make SI control more difficult and pose significant challenges to public health security.

## 2. Materials and Methods

### 2.1. Virus Isolation and Identification

Nasal swab samples were collected from 100 pigs with suspected SI infection on a farm in Guangdong Province. Each swab was placed in a centrifuge tube containing phosphate‐buffered saline (PBS) supplemented with 2000 U/mL penicillin and 2000 U/mL streptomycin, transported to the laboratory on ice packs, and stored at −80°C. Samples were screened by RT‐qPCR, and positive specimens were selected for virus isolation. The positive samples were centrifuged at 8000 × *g* for 10 min at 4°C, and the supernatant was filtered through a 0.45 μm membrane. The filtrate was inoculated into the allantoic cavity of 9–11‐day‐old specific‐pathogen‐free (SPF) embryonated chicken eggs and incubated at 37°C for 72 h. Allantoic fluid was harvested and tested for influenza virus using a HA assay with 0.5% chicken red blood cells. Viral RNA was extracted using the RNeasy Kit (Qiagen, Germany), and the isolate was subtyped by reverse transcription polymerase chain reaction (RT‐PCR) with subtype‐specific primers.

### 2.2. RT‐PCR Amplification and Sequencing

RNA was extracted from the harvested allantoic fluid using the RNeasy Kit (Qiagen, Germany). Eight pairs of primers targeting the complete viral genome were designed based on H1N1 reference sequences from GenBank and synthesized by Sangon Biotech (Shanghai, China). For the polymerase genes (PA, PB1, and PB2), the full‐length sequences were amplified by splitting each gene into two fragments. (Table S1). Reverse transcription and PCR amplification were conducted using the one step RT‐PCR Kit (TOROIVD, Shanghai). PCR products were confirmed by 1% agarose gel electrophoresis and submitted to Sangon Biotech (Shanghai, China) for sequencing.

### 2.3. Homology and Phylogenetic Analysis

Sequences were assembled and edited using Lasergene SeqMan (DNAStar, Madison, WI, USA). Homology searches were conducted using the NCBI BLASTn algorithm against both the NCBI nucleotide collection and GISAID EpiFlu databases, evaluating both sequence identity and query coverage to ensure accurate alignment and avoid potential bias. Reference sequences were retrieved from GenBank, and phylogenetic trees were constructed in MEGA 7.0 using the neighbor‐joining method with 1000 bootstrap replicates. Key amino acid substitutions were analyzed using Lasergene MegAlign (DNAStar, Madison, WI, USA).

Reference sequences for phylogenetic reconstruction were selected based on three strictly defined criteria: (1) the top BLASTn hits were included to identify the closest genetic relatives; (2) representative strains from established global SIV lineages (CS H1N1, EA H1N1, pdm/09 H1N1, TR H1N2, and human H1N1) were selected to facilitate accurate lineage assignment; and (3) temporally and geographically relevant strains circulating in China were included to contextualize local evolutionary dynamics.

### 2.4. Determination of Mouse Lethal Dose 50% and Pathogenicity Study

Healthy 6‐week‐old female BALB/c mice were divided into six groups (11 mice per group). One group served as the negative control, and the remaining five groups were inoculated with the influenza virus at five different titers: 10^5^, 10^4^, 10^3^, 10^2^, and 10^1^ EID_50_/50 μL. After anesthesia with tribromoethanol, each mouse received 50 μL of viral suspension intranasally using a 100 μL micropipette, alternating between nostrils. Mice were then placed in a warm environment to recover from anesthesia. On days 3 and 5 postinfection, three mice from each group were euthanized, and the spleen, kidneys, brains, lung, and nasal turbinate tissues were collected for viral titration in SPF embryonated chicken eggs. The remaining mice were monitored daily for 14 days to record their body weight, clinical signs, and mortality. Body weights were measured daily, and data were normalized to the initial weight of each mouse on day 0 (set as 100%) to calculate the daily percentage of initial body weight. The MLD_50_ was calculated using the Reed–Muench method based on cumulative mortality.

### 2.5. Piglet Experiments

Eighteen 8‐week‐old healthy, susceptible pigs (negative for SIV antigen with HA inhibition (HI) antibody titers <1:10) were selected. Fourteen pigs were randomly assigned to the infection group and inoculated with 4.0 mL of viral suspension (10^4.67^ EID_50_/50 μL per animal) through a tracheal injection. The remaining four pigs received 4.0 mL of sterile PBS using the same route and were housed under identical isolation conditions as negative controls. Body temperature and clinical signs were monitored daily from days 1–21 postinoculation. Nasal swabs and serum samples were collected on days 1, 3, 5, 7, 9, 14, and 21. To evaluate viral shedding, supernatants from nasal swabs were serially diluted and inoculated into SPF embryonated chicken eggs to determine the EID_50_. On days 3, 5, and 7 postinoculation, three pigs were euthanized each day for necropsy to assess lung lesions and determine the severity of pathology [24].

### 2.6. Histopathological Examination

Lung tissues collected from euthanized mice and piglets were fixed in 10% phosphate‐buffered formalin, dehydrated, embedded in paraffin, and sectioned. Tissue sections were stained with hematoxylin and eosin, and pathological changes were examined under a light microscope.

### 2.7. HI Tests

Serum samples from the mouse and piglet experiments were analyzed for antibody levels. Samples were pretreated to remove nonspecific HI and then mixed with 4 HAU of viral antigen, followed by the addition of 0.5% chicken red blood cell suspension. A HI titer greater than 1:10 was considered positive.

### 2.8. Statistical Analysis

Statistical analyses were conducted using GraphPad Prism 5.0 (GraphPad Software Inc., San Diego, CA, USA). Viral titers in mouse tissues were compared using a two‐way ANOVA. Mouse survival data were analyzed using the log‐rank (Mantel–Cox) and Gehan–Breslow–Wilcoxon tests. A *p*‐value less than 0.05 was considered statistically significant.

## 3. Results

### 3.1. Virus Isolation and Identification

Among the collected specimens, 68 samples tested positive for influenza A virus nucleic acid by RT‐qPCR. Six samples exhibiting high viral loads (Ct <26) were selected for virus isolation. The filtrate from these samples was inoculated into the allantoic cavity of 9–11‐day‐old SPF embryonated chicken eggs and incubated at 37°C for 72 h. The harvested allantoic fluid was subjected to an HA assay. Isolates showing HA activity were purified through three consecutive rounds of limiting dilution, yielding a purified SIV strain. Subtyping by RT‐PCR with type‐specific primers identified the isolates as an H1N1 SIV (Figure 1A). Transmission electron microscopy (TEM) revealed spherical or elliptical viral particles ~100 ± 20 nm in diameter, an envelope containing numerous radially arranged surface projections characteristic of influenza viruses (Figure 1B). Following standard nomenclature guidelines, the isolate was designated A/Swine/Guangdong/JJK/2025(H1N1), abbreviated as the SIV H1N1 JJK strain, and reached a 50% egg infectious dose (EID_50_) of 10^6.5^/mL and a 50% tissue culture infectious dose (TCID_50_) of 10^5.7^/mL in MDCK cells. The nucleotide sequences of the JJK strain genome have been deposited in GenBank under Accession Numbers PX936485‐PX936492.

Figure 1Identification of the A/swine/Guangdong/JJK/2025 strain. (A) Identification of the isolate by RT‐PCR using specific primers of the HA and NA genes. (B) Transmission electron microscopy (TEM) image showing typical orthomyxovirus morphology by negative staining (bar = 100 nm). (C) Whole‐genome identification by RT‐PCR using segment‐specific primers for all eight gene segments.

**Figure:**
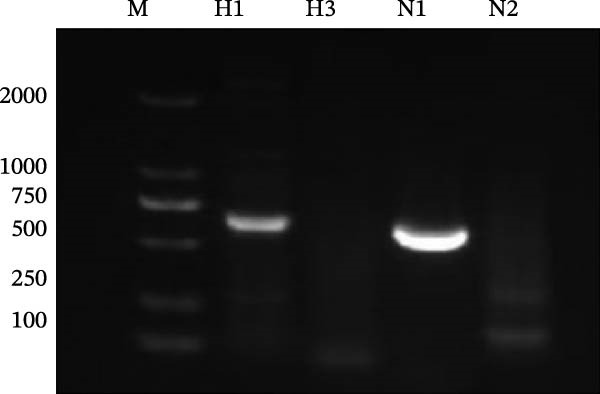
(A)

**Figure:**
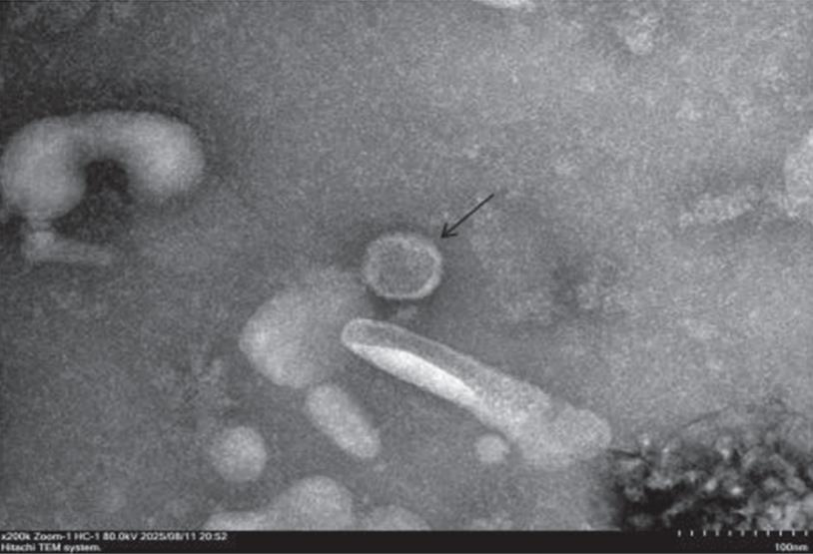
(B)

**Figure:**
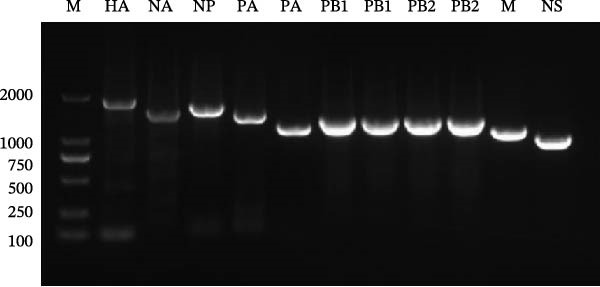
(C)

### 3.2. Homology Analysis

To characterize the genetic evolutionary features of the virus, all eight genomic segments of the SIV H1N1 JJK strain were sequenced (Figure 1C). NCBI BLAST analysis revealed that each segment displayed the highest nucleotide homology to a different reference influenza virus strain. The HA gene shared 97.19% identity with A/Swine/Beijing/F3/2022(H1N1) detected in Beijing in 2022. The NA, PB1, PB2, and PA genes showed highest homology to A/Swine/Liaoning/TL5239/2020(H1N1) (97.44%), A/Swine/Liaoning/TL5404/2020(H1N1) (96.20%), A/Swine/Liaoning/JZ164/2020(H1N1) (97.74%), and A/Swine/Liaoning/AS1732/2020 (H1N1) (97.67%), respectively, all isolated in Liaoning in 2020. The NP gene showed 97.52% identity with A/Swine/Hebei/0221/2017(H1N1), the NS gene shared 98.69% identity with A/Swine/Jiangsu/HD11/2020(H1N1), and the M gene exhibited 99.08% identity with A/Swine/China/SD6591/2019(H1N1). Furthermore, all gene segments exhibited 100% query coverage when aligned with their respective reference strains showing the highest sequence identity (Table 1).

**Table 1:** Influenza virus strains in GenBank showing the highest nucleotide homology with each gene segment of A/Swine/Guangdong/JJK/2025(H1N1).

Virus	Gene	Virus with highest similarity	Homology (%)	Coverage (%)
A/Swine/Guangdong/JJK/2025 (H1N1)	HA	A/Swine/Beijing/F3/2022(H1N1)	97.19	100
	NA	A/Swine/Liaoning/TL5239/2020(H1N1)	97.44	100
	PB1	A/Swine/Liaoning/TL5404/2020(H1N1)	96.20	100
	PB2	A/Swine/Liaoning/JZ164/2020(H1N1)	97.74	100
	PA	A/Swine/Liaoning/AS1732/2020(H1N1)	97.67	100
	NP	A/Swine/Hebei/0221/2017(H1N1)	97.52	100
	NS	A/Swine/Jiangsu/HD11/2020(H1N1)	98.68	100
	M	A/Swine/China/SD6591/2019(H1N1)	99.08	100

### 3.3. Phylogenetic Analysis

**Figure 2:**
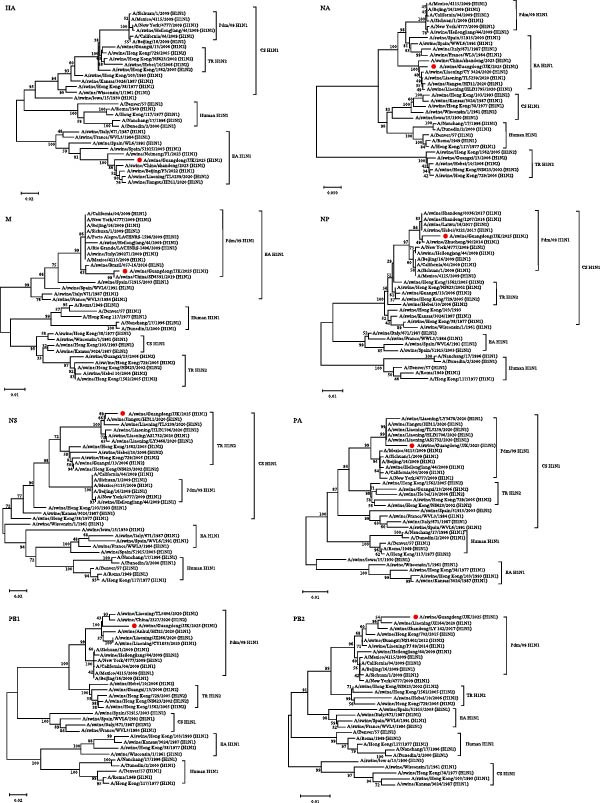
Phylogenetic analysis of all eight gene segments was conducted using the neighbor‐joining method in MEGA 7. Node reliability was assessed using 1000 bootstrap replicates. The viruses isolated in this study are marked with solid red circles.

Eight phylogenetic trees were constructed based on the eight genomic segments to characterize the evolutionary relationships of the SIV H1N1 JJK strain (Figure 2). The HA and NA genes clustered within the EA H1N1 lineage. The internal genes (PB2, PB1, PA, NP, and M) originated from the 2009 pandemic H1N1 (pdm/09 H1N1) lineage, whereas the NS gene belonged to the triple‐reassortant H1N2 (TR H1N2) lineage. These findings confirm that the isolate is a triple‐reassortant H1N1 virus.

### 3.4. Molecular Features Analysis

Amino acid sequence analysis identified the HA cleavage site protein as PSIQSR/GLF, which lacks a polybasic cleavage motif and is consistent with low‐pathogenicity influenza viruses. Additional substitutions in HA included A158G, 190D, and 225E. No resistance‐associated mutations—E119V, R152K, H275Y, R293K, or N295S—were detected in the NA protein.

In the PB2 protein, substitutions 251K, T271A, 588I, and A591S were observed. The PB1 protein contained the substitutions R198K, N375S, H436Y, L473V, and L598P. The PA protein exhibited P224S, L295P, 336M, 356R, 409N, and A515T. Substitutions K184A and Q357K were identified in the NP protein. The M protein contained A30S and S31V, and the NS protein exhibited P42S, G149A, and K186E (Table S2).

### 3.5. Pathogenicity in Mice

#### 3.5.1. Weight Changes and Survival Rates in Mice

Mice are widely used as mammalian models for assessing the pathogenicity of influenza viruses. In this study, each female BALB/c mouse was inoculated intranasally with 50 μL of diluted virus suspension. Following inoculation, mice in the challenged groups developed varying degrees of clinical illness, including anorexia, reduced water intake, weight loss, lethargy, dyspnea, ruffled fur, hunched posture, decreased activity, and mild tremors.

Body weight changes are shown in Figure 3A. Mice in the 10^5^ EID_50_/50 μL, 10^4^ EID_50_/50 μL, and 10^3^ EID_50_/50 μL groups exhibited significantly lower body weights than the controls, beginning on day 2. Mice in the 10^5^ EID_50_/50 μL group experienced more than 25% weight loss starting on day 3, with a maximum loss of 29.46%; all mice in this group exceeded the 25% threshold by day 6. In the 10^4^ EID_50_/50 μL and 10^3^ EID_50_/50 μL groups, weight loss greater than 25% occurred on days 6 and 7, respectively. Body weight in these two groups began to recover by day 8. No significant weight loss was observed in the remaining groups relative to the controls.

Figure 3Pathogenicity of the isolated virus in mice. (A) Body weight changes 14 days postinoculation (dpi). (B) Survival curves indicating viral lethality.

**Figure:**
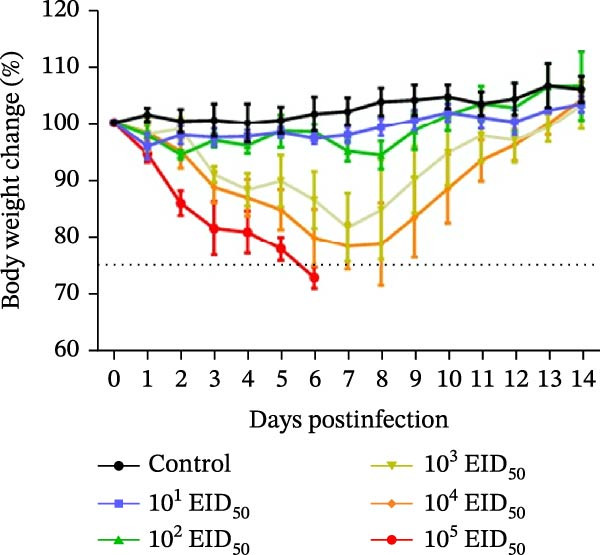
(A)

**Figure:**
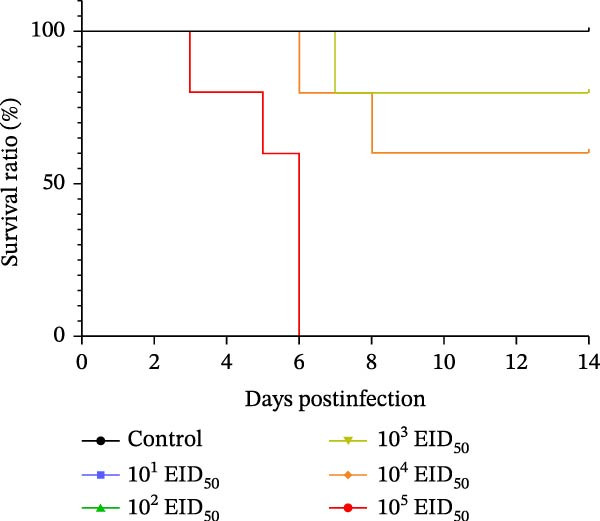
(B)

Using >25% weight loss as the mortality criterion, all mice in the 10^5^ EID_50_/50 μL group died by day 6. In the 10^4^ EID_50_/50 μL group, one mouse died on day 6 and another on day 8, yielding a final survival rate of 60%. In the 10^3^ EID_50_/50 μL group, one mouse died on day 7, resulting in a survival rate of 80%. All mice in the remaining groups survived (Figure 3B).

#### 3.5.2. Viral Titers in Mouse Organs

Organ samples were collected from mice in each group on days 3 and 5 postinoculation to assess viral loads. On day 3, no virus was detected in the spleen, kidneys, or brain. In contrast, the lungs, trachea, and nasal turbinates showed measurable viral titers. The lungs exhibited the highest viral loads, with mean titers ranging from 4.5 to 6.3 log_10_ EID_50_/mL, increasing with higher inoculation doses. Mean viral titers in the trachea and nasal turbinates were 2.2–3.6 log_10_ EID_50_/mL and 3.0–3.4 log_10_ EID_50_/mL, respectively, without a clear dose–response pattern. However, titers in the 10^5^ EID_50_/50 μL group were significantly higher than those in the 10^1^ EID_50_/50 μL group. By day 5, the distribution of viral loads across organs remained similar to that on day 3, with the lungs consistently showing the highest titers. The 10^5^ EID_50_/50 μL group maintained the highest titers, whereas the 10^1^ EID_50_/50 μL group exhibited the lowest titers. Overall titers decreased compared to day 3. Lung, trachea, and nasal turbinate titers measured 4.5–5.8 log_10_ EID_50_/mL, 1.2–3.4 log_10_ EID_50_/mL, and 2.4–3.2 log_10_ EID_50_/mL, respectively (Figure 4A,B).

Figure 4Viral loads in tissues of infected mice. (A) Viral loads in various organs at 3 dpi. (B) Viral loads in various mouse organs at 5 dpi.

**Figure:**
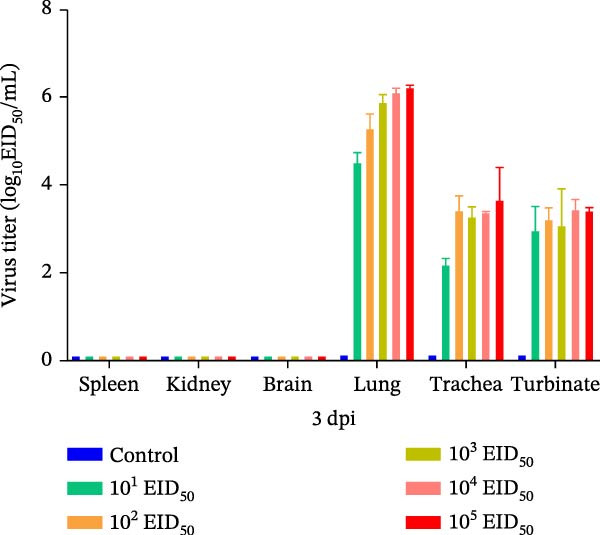
(A)

**Figure:**
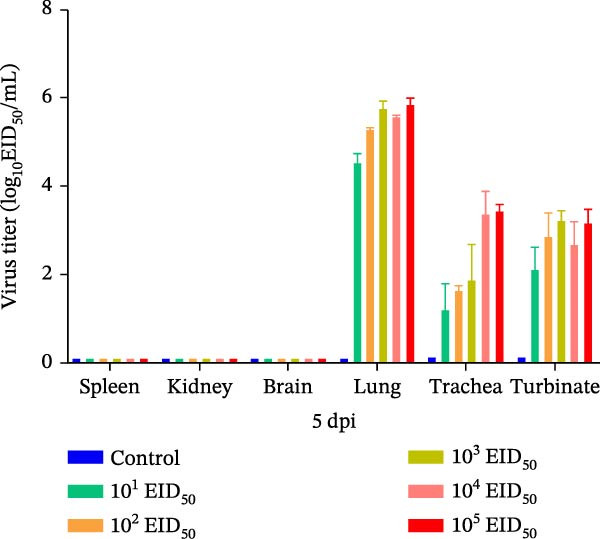
(B)

#### 3.5.3. Gross Lung Lesions and Histopathology in Mice

To correlate viral virulence with pathological outcomes, lung tissues were collected on days 3 and 5 postinoculation for gross examination and histopathological assessment. Gross examination provided critical macroscopic context for the distribution and severity of lung lesions.

On day 3, the infected mice exhibited clear dose‐dependent gross lesions (Figure 5). No abnormalities were present in the control group. Lung tissues from the 10^1^ and 10^2^ EID_50_/50 μL groups showed no visible lesions. Mild redness and swelling were observed in the 10^3^ EID_50_/50 μL group. Severe lesions were evident in the 10^4^ and 10^5^ EID_50_/50 μL groups, characterized by pronounced congestion, edema, and distinct petechiae or ecchymoses on the lung surface.

Figure 5Lung lesions in mice. (A) Gross lung lesions. (B) Histopathological examination of lung tissues.

**Figure:**
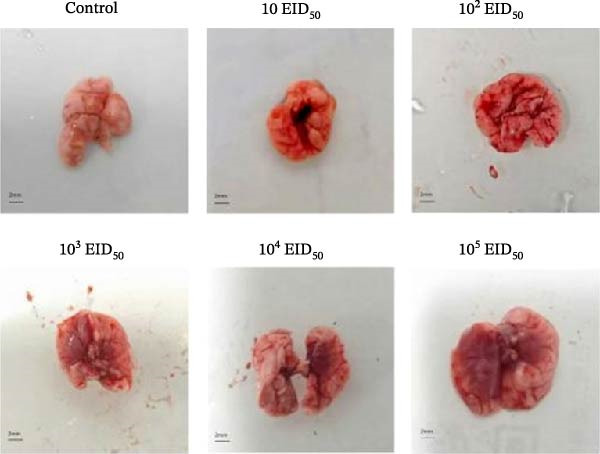
(A)

**Figure:**
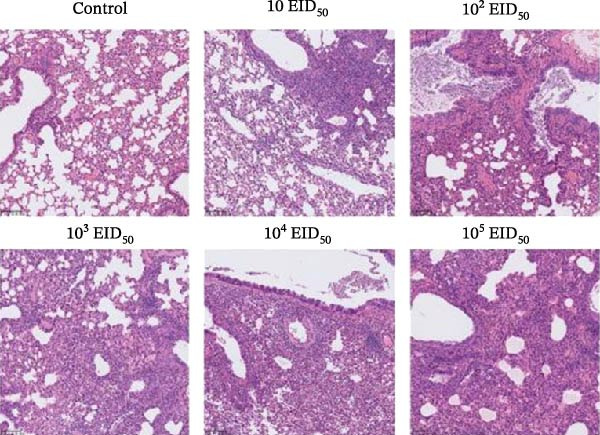
(B)

Histopathological analysis revealed marked tissue damage in the 10^3^, 10^4^, and 10^5^ EID_50_/50 μL groups, including pulmonary consolidation, mild peripheral alveolar dilation, significant alveolar wall thickening, narrowed alveolar spaces, disrupted lung architecture, extensive inflammatory cell infiltration, and focal mild hemorrhage. Some bronchial lumens contained inflammatory exudate and showed mild foam cell hyperplasia. The 10^1^ and 10^2^ EID_50_/50 μL groups displayed mild lesions, including slight peripheral alveolar dilation, alveolar wall thickening, architectural disorganization, moderate inflammatory infiltration, and focal mild hemorrhage. No notable histopathological changes were observed in the control group.

#### 3.5.4. HI Titers of Sera Collected From Mice

Mice were euthanized at 14 days postinoculation (dpi), and serum samples were collected for HI assays to assess antibody responses. All infected groups were seroconverted by day 14. HI titers increased with higher inoculation doses: HI antibody titers ranged from 40 to 160 across all inoculated groups. Specifically, titers in the 10^5^ EID_50_ group ranged between 80 and 160, while the 10^4^ EID_50_ group exhibited a consistent titer of 160. The 10^3^ EID_50_ group showed titers of 80–160. In the lower dosage groups, the 10^2^ EID_50_ group had a titer of 80, and the 10^1^ EID_50_ group ranged from 40 to 80. No antibodies were detected in the control group (Table S3).

### 3.6. Pathogenicity in Piglets

#### 3.6.1. Temperature and Weight Changes in Piglets

To evaluate the pathogenicity of the SIV JJK H1N1 strain in piglets, 8‐week‐old animals were anesthetized and inoculated through the tracheal route with 4 mL of viral suspension containing 10^4.67^ EID_50_/50 μL per pig. Following inoculation, a subset of piglets displayed mild clinical signs, including coughing, nasal discharge, and depression. Body temperature changes are shown in Figure 6A. One piglet exhibited temperatures exceeding 40°C on days 3, 5, 12, 13, and 14 postinoculation, and occasional transient increases were noted in several others. However, overall temperature differences between the infected and control groups (*p* > 0.05) (Figure 6A). Regarding weight gain (Figure 6B), control piglets gained more weight than infected piglets from days 7 to 14 postinoculation, although the difference was not significant (*p* > 0.05). By day 21, the weight gain in the infected group had recovered to levels comparable to those of the control group (Figure 6B).

Figure 6Pathogenicity of the isolated virus in piglets. (A) Temperature changes in piglets. (B) Weight changes in piglets.

**Figure:**
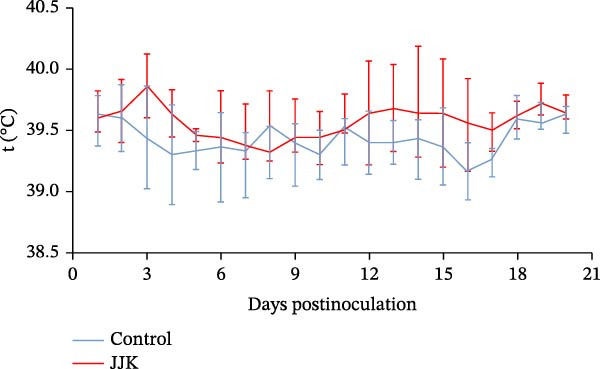
(A)

**Figure:**
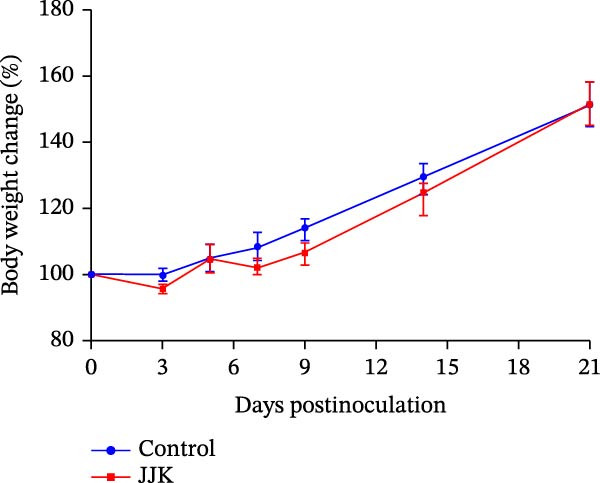
(B)

#### 3.6.2. Viral Shedding Dynamics and Tissue Viral Titers in Piglets

Nasal swabs were collected from infected piglets on days 3, 5, 7, 9, 14, and 21 postinoculation to assess viral shedding. Three piglets from the challenged group were euthanized on days 3, 5, and 7 for necropsy, and lung and tracheal tissues were collected for viral titration. Viral shedding was detectable in nasal swabs from days 3–7 postinoculation, with the highest titers (3–4 log_10_ EID_50_/mL) observed on day 3. Shedding declined steadily over time and became undetectable after day 9 (Figure 7A). Viral titers in the lungs and trachea were also detectable during days 3–7. Peak titers occurred on day 3, reaching ~3 log_10_ EID_50_/mL in the lungs and 2–2.5 log_10_ EID_50_/mL in the trachea. Viral loads in both tissues declined over time, and lung titers were consistently higher than those in the trachea (Figure 7B).

Figure 7Viral titers in samples. (A) Viral titers in nasal swabs from piglets. (B) Viral titers in lung and tracheal tissues from piglets.

**Figure:**
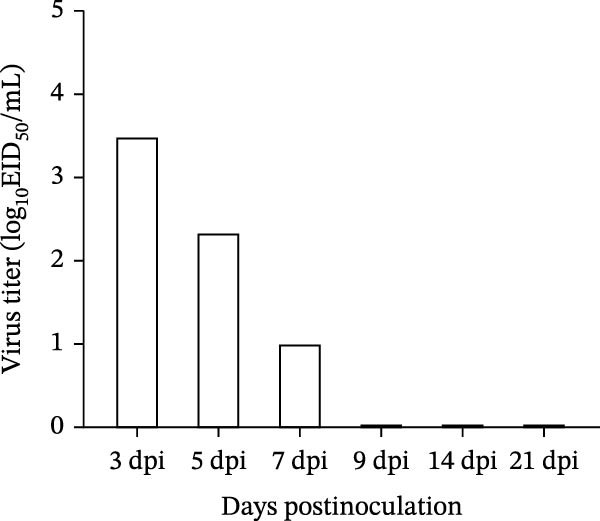
(A)

**Figure:**
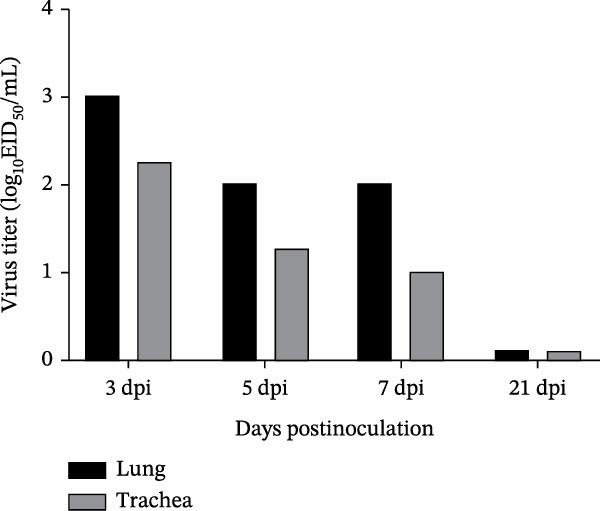
(B)

#### 3.6.3. Lung Pathological Changes and Lesion Scores in Piglets

Gross examination provided critical macroscopic context for the distribution and severity of lung lesions. Piglets in the challenged group exhibited mild lung lesions consistent with influenza virus infection. Grossly, the lungs displayed multifocal to locally extensive red consolidation, primarily distributed in the cranioventral regions, along with abundant mucus accumulation in the trachea (Figure 8A). Histopathological examination revealed marked inflammatory changes compared with the control group, including inflammatory cell aggregation, reduced alveolar numbers, and infiltration of inflammatory cells within alveolar spaces (Figure 8A). Lung lesion severity was quantified using the Wan et al. [24] pneumonia scoring system. The highest scores occurred on 5 day, reaching 240. Lesion scores on 3 day were comparable to those on 5 day. By day 7, lesion severity had decreased notably. Interestingly, on 21 day, the lung lesion score increased relative to that on 7 day (Figure 8B).

Figure 8Lung pathological changes and lesion scores in piglets. (A) Lung pathological changes. (B) Lung lesion scores.

**Figure:**
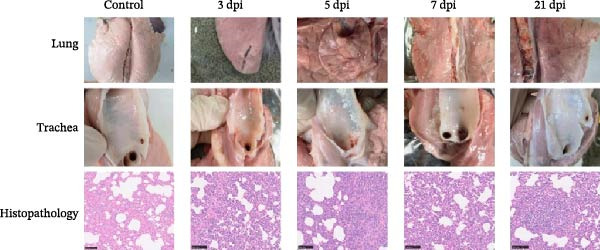
(A)

**Figure:**
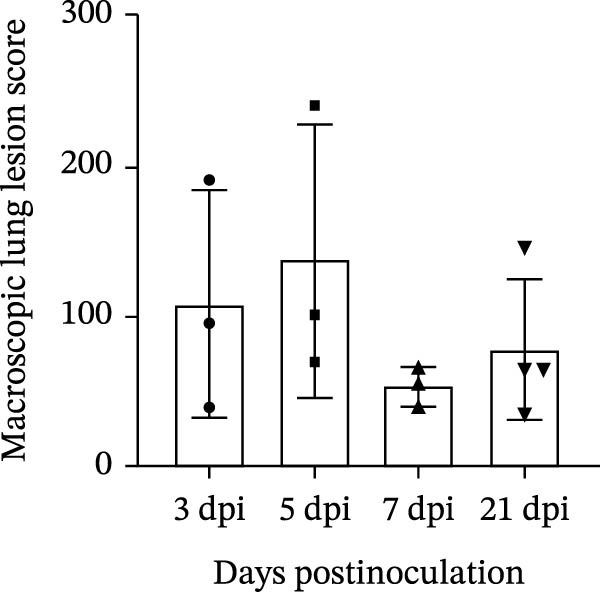
(B)

#### 3.6.4. Serum Inflammatory Cytokines in Piglets

Serum cytokine levels in the challenged group increased in a time‐dependent manner. TNF‐α levels were significantly higher than those in the control group on days 14 and 21 postinoculation (*p* < 0.001), reaching a peak of 122.8 pg/mL on day 21. Serum IL‐10 levels were significantly elevated on days 5, 7, and 9 postinoculation (*p* < 0.05) and reached 73.56 pg/mL on day 21, representing a highly significant increase compared with controls (*p* < 0.001). In the challenged group, IFN‐γ and IL‐6 peaked on days 5 and 7 postinoculation at 41.1 pg/mL and 95.4 pg/mL, respectively. Both cytokines showed highly significant elevations relative to the control group (*p* < 0.001). After reaching their peaks, serum IFN‐γ and IL‐6 concentrations declined as the infection progressed (Figure 9).

Figure 9Serum inflammatory cytokines in piglets. (A) TNF‐α concentration. (B) IFN‐γ concentration. (C) Serum IL‐10 concentration. (D) IL‐6 concentration.

**Figure:**
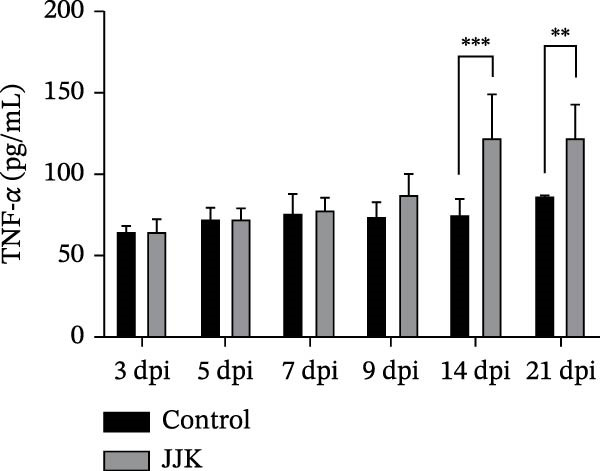
(A)

**Figure:**
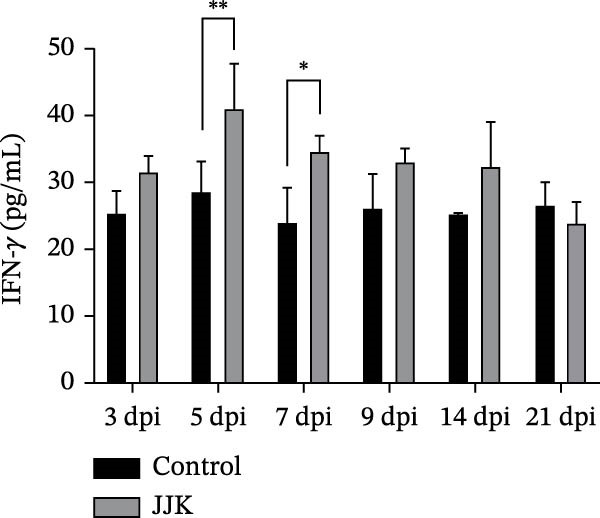
(B)

**Figure:**
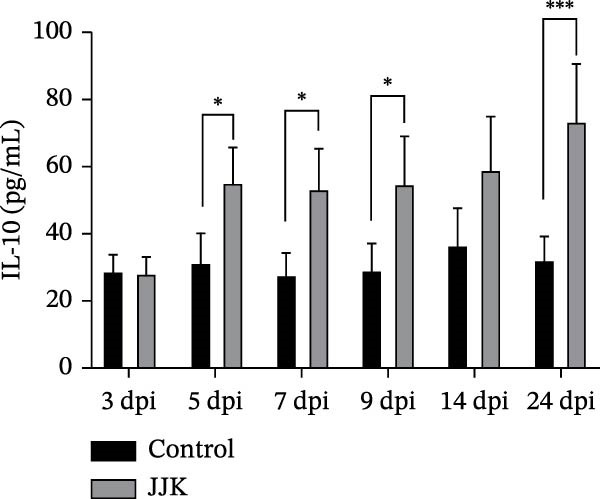
(C)

**Figure:**
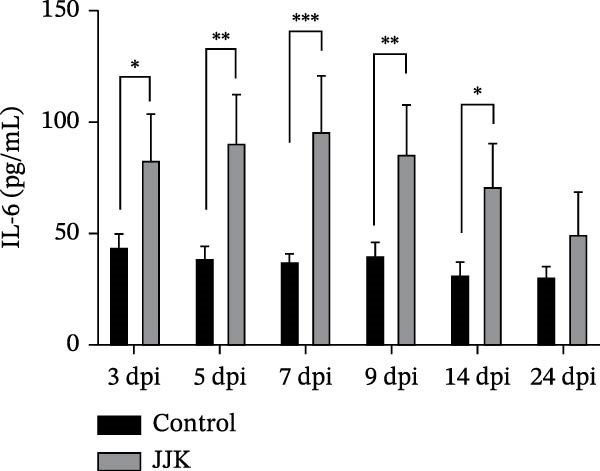
(D)

#### 3.6.5. HI Titers Piglet Serum

Serum samples collected from euthanized piglets on days 7, 14, and 21 postinoculation were analyzed using a HI assay. All challenged piglets seroconverted, and antibody titers increased over time. HI titers ranged from 10 to 40 on 7 day, increased to 20–160 on 14 day, and reached 40–320 on 21 day (Table 2).

**Table 2:** HI antibody titers in piglet serum on days 7, 14, and 21 postinoculation.

dpi	Control			Challenged group							
	E1	E2	E3	F2	F4	F7	F8	F9	F11	F12	F13
7	＜10	＜10	＜10	10	10	10	10	40	20	20	40
14	＜10	＜10	＜10	40	40	20	80	160	—	—	—
21	＜10	＜10	＜10	40	80	80	80	320	—	—	—

## 4. Discussion

SI is an acute zoonotic infectious disease that is widely prevalent in pig populations. It causes respiratory damage in infected pigs, predisposes them to secondary infections, and leads to substantial economic losses in the swine industry. The disease also carries important public health implications [12, 25]. Pigs serve as “mixing vessels” for influenza viruses because the segmented influenza genome enables reassortment, and coinfections provide the conditions necessary for generating novel viral strains. This mechanism underlies the frequent emergence of new influenza variants [26].

In this study, an H1N1 subtype SIV (JJK) exhibiting a triple‐reassortant genotype was isolated from a pig farm in Guangdong using SPF embryonated chicken eggs. Genomic and phylogenetic analyses demonstrated that the JJK strain is a typical triple‐reassortant virus. Its HA and NA genes originate from the EA H1N1 lineage, consistent with surveillance reports showing that EA H1N1 has replaced CS H1N1 as the predominant lineage in Chinese swine herds [27]. Its internal genes (PB2, PB1, PA, NP, and M) are derived from the pdm/09 H1N1 lineage, and its NS gene originates from the TR H1N2 lineage. This genomic constellation—EA surface genes combined with a pdm/09 internal gene backbone—has become increasingly common in Chinese pigs since 2016 [21, 23]. Studies have shown that pdm/09‐derived internal genes can confer adaptive advantages by enhancing viral replication and transmission in mammals, which may facilitate the dominance of such reassortant strains in swine populations [22, 28]. Notably, each of the eight gene segments of the JJK strain showed the highest homology with a different reference strain isolated from a different region and year. This mosaic genomic structure underscores the complexity of SIV evolution in China and reinforces the role of pigs as mixing vessels that facilitate reassortment among viruses of diverse geographical origins and genetic backgrounds.

Molecular characterization is essential for evaluating the potential risks associated with emerging viruses. We identified multiple amino acid substitutions that have been previously reported to correlate with enhanced mammalian adaptation, increased virulence, and modulation of host immune responses. Amino acid analysis of the SIV H1N1 JJK strain revealed several mutations across multiple proteins associated with host adaptation, virulence, and drug susceptibility. The HA cleavage site (PSIQSR/GLF) lacked a polybasic motif, consistent with the molecular features of low‐pathogenicity influenza viruses [29]. Substitutions in the HA receptor‐binding region—A158G, 190D, and 225E—were also identified; these residues are linked to increased binding affinity for SAα‐2,6‐Gal receptors [30–33].

Within the polymerase complex, multiple substitutions were detected in the PB2 protein—including 251K, T271A, 588I, and A591S—all of which can enhance virus replication [34]. Among these, T271A is associated with increased transmission in ferret models [33], whereas 588I and A591S contribute cooperatively to mammalian adaptation [35]. Although the well‐known E627K and D701N substitutions were absent in this strain, both mutations have been documented to enhance pathogenicity in mice [36, 37]. The PB1 protein contained several substitutions associated with increased virulence—R198K, N375S, H436Y, L473V, and L598P [38]. In the PA protein, mutations included P224S, which enhances virulence [39]; L295P, associated with increased viral replication [40]; and 336M, 356R, 409N, and A515T, all linked to heightened pathogenicity in mice [41].

No NA inhibitor resistance‐associated mutations—such as E119V, R152K, H275Y, R293K, or N295S—were detected in the NA protein, indicating that the strain is likely sensitive to NA inhibitors such as oseltamivir [41, 42]. The NP protein contained K184A, a mutation associated with increased virulence and replication capacity [43], and Q357K, which enhances polymerase activity, replication efficiency, and pathogenicity in mice [44]. In the M2 protein, substitutions A30S and S31V were identified, both of which confer resistance to adamantane drugs [45], whereas T215A is associated with increased pathogenicity in mice [46]. The NS1 protein contained P42S and K186E, both of which enhance interferon antagonism, and G149A, a mutation linked to increased viral virulence [47]. These molecular features collectively form the pathogenic basis of the SIV H1N1 JJK strain and are consistent with its observed pathogenicity in mice. We noted that the JJK strain carries HA mutations (A158G, 190D, 225E) associated with human‐type receptor binding and pdm/09 internal genes that facilitate mammalian replication, suggesting a potential risk for cross‐species transmission and emphasizing the need for One Health surveillance.

Our molecular analysis identified numerous signatures associated with high virulence and adaptation. While these markers provide a theoretical basis for the observed pathogenicity in mice, we acknowledge that their functions are inferred from historical data. Since influenza virus proteins function through complex epistatic interactions, the actual contribution of these substitutions to the JJK phenotype remains to be confirmed through reverse genetics and site‐directed mutagenesis in future studies.

Animal pathogenicity studies have supported the functional significance of these molecular characteristics. In the mouse model, the JJK strain caused severe disease. Infection with a high dose (10^5^ EID_50_/50 μL) resulted in rapid weight loss and 100% mortality. Histopathological examination revealed severe diffuse alveolar damage and consolidation. These findings are consistent with reports that EA H1N1 viruses carrying pdm/09‐derived internal genes often display a strong replication capacity and pronounced pathogenicity in mice [48]. Although no virus was detected in mouse brain tissues, the high viral titers in the lungs produced marked pathological injury. The level of respiratory replication alone was sufficient to induce fatal pulmonary damage [39].

In contrast to the mouse model, the JJK strain caused only mild clinical signs in piglets, including slight fever, coughing, and limited pulmonary inflammation, with no mortality observed. Such subclinical presentations are commonly reported in field cases of SIV infection [49]. However, the absence of overt symptoms does not equate to low epidemiologic risk. Infected piglets shed the virus through nasal secretions for up to 9 days, indicating that infected animals may function as silent sources of transmission. Under intensive farming conditions, these “silent shedders” can be easily overlooked, enabling sustained viral circulation within swine herds and potentially increasing the risk of zoonotic spillover [50]. Despite the mild clinical presentation, serum cytokine analyses revealed significant elevations in IL‐6, TNF‐α, and IFN‐γ. As reported by Lee et al. [51], the early induction of cytokines such as IFN‐α, TNF‐α, and IL‐6 following SIV infection correlates strongly with fever and pulmonary inflammation. This robust inflammatory response may contribute to immunopathological injury, providing a plausible explanation for the mild clinical signs observed in piglets despite measurable lung lesions.

The discrepancy between severe murine pathology and mild clinical signs in piglets may stem from host‐specific differences in receptor distribution or cytokine regulation. The stark contrast in clinical outcomes between the murine and porcine models may be attributed to differences in receptor distribution and host‐specific immune responses. While the identified HA mutations (A158G, 190D, and 225E) enhance binding to the mammalian SAα‐2,6‐Gal receptor [30–33] murine models are often hypersensitive to high‐dose intranasal inoculation, where deep lung deposition can trigger rapid, fatal viral pneumonia. In contrast, pigs serve as the natural host with a respiratory tract containing both SAα‐2,6 and SAα‐2,3 receptors. The robust elevation of serum cytokines (IL‐6, TNF‐α, and IFN‐γ) observed in the infected piglets suggests an active immune defense that effectively limited systemic viral spread, resulting in subclinical infection despite the presence of lung lesions [51].

## 5. Conclusion

This study characterized the genetic and pathogenicity properties of the triple‐reassortant H1N1 subtype SIV circulating in Guangdong pig populations. The strain exhibits a complex reassortant genomic background and carries multiple molecular markers associated with enhanced mammalian adaptation and virulence. Consistent with these molecular markers, the virus demonstrated significant virulence in the BALB/c mouse model under high‐dose experimental conditions. Although pathogenicity was relatively low in pigs, the virus demonstrated measurable transmissibility within swine herds and presented a potential risk of cross‐species transmission. These findings highlight the need for strengthened, continuous surveillance and comprehensive risk assessment of SIV in Chinese pig populations, providing critical scientific evidence to support disease control and early warning systems for public health protection.

## Author Contributions

Yu Wu and Benkuan Li conceived and designed the study. Benkuan Li and Qunhui Li devised the experimental methods. Yi Song, Hui Guo, and Guangrun Qin curated the data. Qunhui Li, Haishen Zhao, Limiao Lin, Lianxiang Wang, and Bohua Ren provided resources and performed the experiments. Benkuan Li and Yu Wu prepared the original manuscript draft. Lianxiang Wang and Huihua Zhang reviewed the manuscript and edited it.

## Funding

This research was supported by the open competition program of top 10 critical priorities of Agricultural Science and Technology Innovation for the 14th Five‐Year Plan of Guangdong Province (Grant 2024KJ14).

## Disclosure

All authors read and approved the final manuscript.

## Ethics Statement

Our animal experiments were approved by the Animal Ethics Committee of Foshan University and conducted under the guidance of the Foshan University Institutional Animal Care and Use Committee (FOSU#19‐025).

## Conflicts of Interest

The authors declare no conflicts of interest.

## Supporting Information

Additional supporting information can be found online in the Supporting Information section.

## Supporting information


**Supporting Information** Table S1: Primer set used for RT‐PCR amplification of the eight vRNAs of influenza A viruses. Table S2: Molecular characteristics of the isolated virus strain. Table S3: HI antibody titers in mouse serum at 14 days postinoculation (dpi).

## Data Availability

The experimental data used to support the findings of this study are available from the corresponding author upon request.
