# Supplementary material for: Genetic Characterization and Pathogenicity of a Triple‐Reassortant H1N1 Swine Influenza Virus Isolated in Guangdong
Source: Transbound Emerg Dis. 2026 Apr 10;2026:7175378. doi: 10.1155/tbed/7175378 (PMC13069155; doi:10.1155/tbed/7175378)
Supplement: Supplementary file 1 — Supporting Information Table S1: Primer set used for RT‐PCR amplification of the eight vRNAs of influenza A viruses. Table S2: Molecular characteristics of the isolated virus strain. Table S3: HI antibody titers in mouse serum at 14 days postinoculation (dpi). [file TBED-2026-7175378-s001.docx]

| Gene | Forward primer | Reverse primer | Expected Size (bp) |
| --- | --- | --- | --- |
| HA | TATTCGTCTCAGGGAGCAAAAGCAGGGG | ATATCGTCTCGTATTAGTAGAAACAAGGGTGTTTT | 1778 |
| NA | TATTCGTCTCAGGGAGCAAAAGCAGGAGT | ATATCGTCTCGTATTAGTAGAAACAAGGAGTTTTT | 1565 |
| NP | TATTCGTCTCAGGGAGCAAAAGCAGGGTA | ATATCGTCTCGTATTAGTAGAAACAAGGGTATTTTT | 1413 |
| M | TATTCGTCTCAGGGAGCAAAAGCAGGTAG | ATATCGTCTCGTATTAGTAGAAACAAGGTAGTTTTT | 1027 |
| NS | TATTCGTCTCAGGGAGCAAAAGCAGGGTG | ATATCGTCTCGTATTAGTAGAAACAAGGGTGTTTT | 890 |
| PA-1 | TATTCGTCTCAGGGAGCGAAAGCAGGTCA | ACATCCTGTTTTCTTCCTTCTGCCTTT | 1532 |
| PA-2 | TAATGGGCCACTTTGCCATCAGC | ATATCGTCTCGTATTAGTAGAAACAAGGTACTT | 1283 |
| PB1-1 | TATTCGTCTCAGGGAGCGAAAGCAGGCA | ATATGACCACCCTACCCGAGGTTAGTA | 1332 |
| PB1-2 | TAATTCGCAAGACACAGAAATTTCCTTCAC | ATATCGTCTCGTATTAGTAGAAACAAGGCATTT | 1351 |
| PB2-1 | TATTGGTCTCAGGGAGCGAAAGCAGGTC | ATAAGAGTGTTCTCCTAACGTACTATTTCCGT | 1338 |
| PB2-2 | TATGCAAGGCTGCAATAGGGTTGAGAATT | ATATGGTCTCGTATTAGTAGAAACAAGGTCGTTT | 1356 |

Table S1. Primer set used for RT-PCR amplification of the eight vRNAs of influenza A viruses

Table S2. Molecular characteristics of the isolated virus strain

| Gene | Mutational Site | Putative Function(Based on Literature) | Site | References |
| --- | --- | --- | --- | --- |
| HA | PSIQSR/GLF | Cleavage site |  | [29] |
|  | A158G | May enhance binding to human-type SAα-2,6 receptors, Potential modulation of receptor binding affinity | G | [30-33] |
|  | 190D |  | D |  |
|  | 225E |  | E |  |
| PB2 | 251K | Linked to increased viral replication | K | [34] |
|  | T271A | Linked to increased transmission in ferrets | A | [33] |
|  | 588I | Linked to increased adaptation in mammalian | I | [34] |
|  | A591S |  | S |  |
| PB1 | R198K | Putative contribution to enhanced pathogenicity | K | [38] |
|  | N375S | Putative contribution to enhanced pathogenicity | S | [38] |
|  | H436Y |  | Y |  |
|  | L473V |  | V |  |
|  | L598P |  | P |  |
| PA | P224S | Putative contribution to enhanced pathogenicity | S | [39] |
|  | L295P | Implicated in enhanced replication of the virus | P | [40] |
|  | 336M | Linked to increased virulence in murine models | M | [41] |
|  | 356R |  | R |  |
|  | 409N |  | N |  |
|  | A515T | Linked to increased virulence in murine models | T | [41] |
| NA | E119V | Neuraminidase inhibitor resistance | E | [41,，，，[42] |
|  | R152K |  | R |  |
|  | H275Y |  | H |  |
|  | R293K |  | R |  |
|  | N295S |  | N |  |
| NP | K184A | Putative contribution to enhanced pathogenicity | A | [43] |
|  | Q357K | Implicated in enhanced polymerase activity, replication | K | [44] |
| M | A30S | Implicated in enhance amantadine drug resistance | S | [45] |
|  | S31V |  | V |  |
| NS | P42S | Implicated in enhance the drug resistance of IFN | S | [47] |
|  | G149A | Putative contribution to enhanced pathogenicity | A | [47] |
|  | K186E | Implicated in enhance the drug resistance of IFN | E | [47] |

Table S3. HI antibody titers in mouse serum at 14 days post-inoculation (dpi)

| Group | Control | | | | | 10^1^EID_50_ | | | | | 10^2^EID_50_ | | | | | 10^3^EID_50_ | | | | | 10^4^EID_50_ | | | | | 10^5^EID_50_(6dpi) | | | | |
| --- | --- | --- | --- | --- | --- | --- | --- | --- | --- | --- | --- | --- | --- | --- | --- | --- | --- | --- | --- | --- | --- | --- | --- | --- | --- | --- | --- | --- | --- | --- |
| Number | 1 | 2 | 3 | 4 | 5 | 1 | 2 | 3 | 4 | 5 | 1 | 2 | 3 | 4 | 5 | 1 | 2 | 3 | 4 | 5 | 1 | 2 | 3 | 4 | 5 | 1 | 2 | 3 | 4 | 5 |
| HI titers | 0 | 0 | 0 | 0 | 0 | 80 | 80 | 80 | 40 | 80 | 80 | 80 | 80 | 80 | 80 | 80 | - | 80 | 80 | 160 | - | 160 | 160 | 160 | - | 160 | 160 | 80 | - | - |
